# Genetic analysis of 55 cases with fetal skeletal dysplasia

**DOI:** 10.1186/s13023-022-02559-4

**Published:** 2022-11-09

**Authors:** Ying Bai, Yue Sun, Ning Liu, Li Wang, Zhihui Jiao, Yaqin Hou, Huikun Duan, Qianqian Li, Xiaofan Zhu, Jingjing Meng, Xiangdong Kong

**Affiliations:** Genetic and Prenatal Diagnosis Center, Department of Obstetrics and Gynecology, the First Affiliated Hospital of Zhengzhou University, Zhengzhou, 450052 China

**Keywords:** Skeletal dysplasia, Molecular diagnosis, Genotype–phenotype analysis, Breakpoints, Novel variants

## Abstract

**Background:**

Fetal skeletal dysplasia (SD) is a common congenital disability comprising a complex group of skeletal disorders with substantial clinical and genetic heterogeneity. Many of these defects are detected prenatally using ultrasound (US). However, the diagnostic accuracy of the US is limited.

**Methods:**

We recruited 55 unrelated fetuses with US-detected skeletal anomalies and performed sequential tests using copy number variation sequencing, targeted skeletal gene panel sequencing, or whole exome sequencing. The detected variants were validated using Sanger sequencing or multiplex ligation-dependent probe amplification. We conducted breakpoint analysis and structural modeling of variants possibly involved in fetal SD.

**Results:**

A definitive diagnosis was achieved in 81.82% of affected fetuses (45/55). We identified chromosomal abnormalities in seven cases and 36 variants, of which 18 were novel pathogenic or likely pathogenic in 11 genes in 38 cases. De novo variants were identified in 27 cases (71.05%, 27/38), and one gonosomal mosaicism variant was found in the mother of one fetus. Our case examples demonstrated the high heterogeneity of fetal SDs and the rare fetal SD-associated challenges.

**Conclusions:**

Careful clinical evaluation of fetuses with SD can guide appropriate molecular testing. Our study extends the SD-associated pathogenic variant spectrum and provides useful genetic counselling guidance and an accurate prenatal diagnosis strategy.

**Supplementary Information:**

The online version contains supplementary material available at 10.1186/s13023-022-02559-4.

## Background

Fetal skeletal dysplasia (SD) is one of the most common congenital disabilities in clinical practice. It has been reported that the prevalence of fetal SD is 2.3 to 4.5 per 10.000 births [1]. SD is a group of heterogeneous disorders associated with skeletal abnormalities, including bone shape, size, and density abnormalities, manifesting as malformed limbs, chest, or skull. According to the 2019 nosology and classification of genetic skeletal disorders, there are 461 types of hereditary bone diseases, divided into 42 classes, involving 437 genes [2]. Moreover, skeletal abnormalities may occur in other multisystem syndromes [3]. Due to the high clinical and genetic heterogeneity of SD, it is challenging to make a precise diagnosis of fetal SD.

Although ultrasound (US) is still an indispensable first-line fetal malformation screening method during pregnancy, it has severe limitations owing to the complex and overlapping phenotypes of fetal SD, especially non-fatal fetal SD. The lack of an accurate prenatal diagnosis makes prenatal counselling and pregnancy management challenging. Therefore, the combination of molecular diagnosis with US screening offers the possibility of identifying the cause of SD. In this study, 55 fetuses with SD were recruited and analyzed using copy number variation sequencing (CNV-seq), targeted skeletal gene panel sequencing, or whole exome sequencing (WES). A qPCR walking strategy and long-range PCR were subsequently performed to identify the breakpoint junction of a novel deletion. Then, in silico prediction of the functional impact of the rare novel variant in *GNAI3* was conducted. The present study aimed to enrich the phenotype and genotype of fetal SD, provide a definitive prenatal diagnosis for 55 affected families to improve genetic counselling, reduce the risk of recurrence, and characterize the molecular strategies to diagnose fetuses with SD.

## Methods

### Fetuses and sample collection

This study included 55 fetuses diagnosed with skeletal abnormalities with or without other systemic abnormalities using US at the First Affiliated Hospital of Zhengzhou University between January 2019 and March 2021. The fetuses were not exposed to tobacco, alcohol, radiation, or infectious diseases during pregnancy. We collected fetal abortion tissues (46/55), amniocytes (9/55), and peripheral blood samples from 96 parents. Genomic DNA was obtained using QIAamp DNA Blood Mini Kit 250 (Qiagen, Hilden, Germany) according to standard extraction methods. The present study was approved by the ethics committee (KS-2018-KY-36) of the First Affiliated Hospital of Zhengzhou University, and informed consents were obtained from all the parents of the fetuses.

### Quantitative fluorescent (QF)-PCR and CNV-seq

QF-PCR and CNV-seq were performed as previously described [4, 5]. The identified CNVs were then mapped to the reference genome GRCh37/hg19. According to previously reported guidelines, we interpreted and classified the clinical significance of candidate CNVs using databases of Genomic Variants (http://dgv.tcag.ca/dgv/app/home), Database of Chromosomal Imbalance and Phenotype in Humans Using Ensembl Resources (https://decipher.sanger.ac.uk/), ClinVar (https://www.ncbi.nlm.nih.gov/clinvar/), Online Mendelian Inheritance in Man (OMIM) database (https://omim.org/) and ClinGen (https://www.clinicalgenome.org/).

### Next generation sequencing (NGS)

For fetuses 11–25 (2019.1–2019.6), a special NGS gene panel analysis (including 811 congenital skeletal anomalies genes, Additional file 1: Table S1) was performed by a commercial company (MyGenostics, Inc., Beijing, China). For fetuses 8–10 and 26–55 (2019.7–2021.3), WES was performed and enriched for exonic sequences using the IDT xGen Exome Research Panel V1.0 (Integrated DNA Technologies, San Diego, USA). Sequencing results were mapped to the human genome (GRCh37/hg19). All variants were annotated using databases including the 1000 genomes project (1000G, http://www.internationalgenome.org/), dbSNP (https://www.ncbi.nlm.nih.gov/snp/), the genome aggregation database (GnomAD, https://gnomad.broadinstitute.org/), ClinVar, the Human Genomic Mutation Database (HGMD, http://www.hgmd.cf.ac.uk/ac/index.php) and OMIM. Variants were classified according to the guidelines recommended by the American College of Medical Genetics and Genomics (ACMG).

### Verification of gene variants

Pathogenic or likely pathogenic variants were verified using Sanger sequencing. Multiplex ligation-dependent probe amplification (MLPA) was performed to detect the copy number of the *EVC2* gene in case 49 and family members using the P456 probe mix (MRC-Holland, Amsterdam, The Netherlands) on an ABI PRISM 3100 Genetic Analyzer according to the manufacturer’s instructions. The obtained data were analyzed using the Coffalyser software.

### Breakpoint analysis

To identify the junction of the gross deletion in case 49, a qPCR walking strategy and long-range PCR were performed. Multiple primers were designed to be regularly spaced every ~ 25 kb (step 1), ~ 5 kb (step 2), and ~ 1.5 kb (step 3) within the breakpoint regions (Fig. 4, Additional file 1: Table S2). qPCR analysis was performed using the FastStart Universal SYBR Green Master kit (Roche Applied Science, Germany) in ABI QuantStudio 5 according to the manufacturer’s instructions and the 2^−△△Ct^ method, as previously described [6]. Long-range PCR was performed with the forward primer of D131 and the reverse primer of U4 using Phusion High-Fidelity DNA polymerases (Thermo Scientific, Schwerte, Germany). PCR products were analyzed using electrophoresis in 1% agarose gel, and the subsequent sequences were aligned to the reference genome hg19 using the BLAT tool of the UCSC Genome Browser (https://www.genome.ucsc.edu/) to identify the breakpoint. Finally, the 200 bp reads upstream and downstream of the breakpoints were analyzed to search for repetitive elements using the “RepeatMasker” program (UCSC).

### Conservation analysis and molecular model

Protein sequencing data used in the conservation analysis was downloaded from the NCBI HomoloGene database (https://www.ncbi.nlm.nih.gov/homologene/). The UGENE software was used for the visualization of multiple sequence alignments. A structural model of the novel variant in *GNAI3* was constructed using the homology-modeling server SWISS-MODEL (https://swissmodel.expasy.org/) and visualized using PyMOL software, as previously described [7].

## Results

### Clinical features of cases

Among the 55 families in the present study, the median age of pregnant women was 28 years, and the median gestational age was 23 weeks. According to the clinical characteristics detected using US, fetuses were divided into three groups: 10 (18.18%) had multiple malformations, 41 (74.55%) exhibited short limb deformities (short long bones less than -2standard deviation) with or without long bone bending, fracture, “telephone receiver-shaped” changes, or thoracic hypoplasia, and 4 (7.27%) isolated SD (hypomineralization, premature closure of cranial suture, small mandible, and syndactyly). Detailed information about the clinical features is provided in Additional files 1 and 2: Table S3 and Fig. S1–S12.

### Genetic analysis

**Fig. 1:**
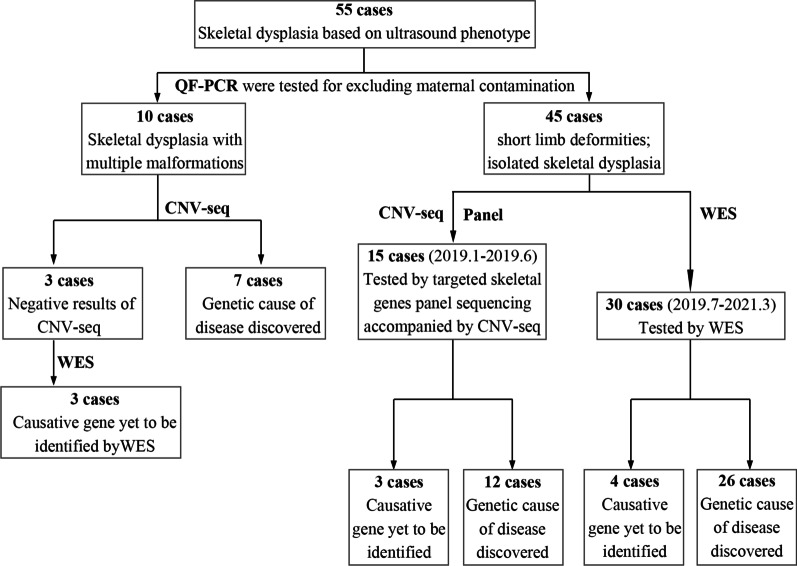
Flowchart showing the methods and results of 55 cases enrolled in our study. QF-PCR: quantitative fluorescent PCR; CNV-seq: copy number variation sequencing; Panel: **a** special NGS gene panel analysis (including 811 congenital skeletal anomalies genes); WES: whole exome sequencing

A total of 55 fetuses with suspected SD were studied, and 45 cases were diagnosed with hereditary diseases, with a diagnostic rate of 81.82% (45/55) (Fig. 1, Table 1). CNV-seq was performed for 1–10 fetuses with multiple malformations, and chromosomal abnormalities were identified in seven fetuses (70%, 7/10), including cases 1–5 with trisomy-18, case 6 with pathological microdeletions and microduplications (Xp22.33p22.12(2700000–19680000) × 1; 11p15.5p15.4 (180000–7100000) × 3; 20q13.2 (50380000–50640000) × 1), and case 7 with triploidy (69-XXX).

**Table 1:** Summary of main malformation classification and molecular results for 55 cases with skeletal abnormalities

NO.	G (weeks)	Main malformation classification	Method	Molecular result	Inheritance	Variant type (ACMG)
1	20^+5^	Multiple malformations	CNV-seq	Trisomy-18		
2	13^+^	Multiple malformations	CNV-seq	Trisomy-18		
3	15	Multiple malformations	CNV-seq	Trisomy-18		
4	18^+2^	Multiple malformations	CNV-seq	Trisomy-18		
5	32^+^	Multiple malformations	CNV-seq	Trisomy-18		
6	23^+^	Multiple malformations	CNV-seq	46, XX; Xp22.33p22.12		
				(2700000-19680000) x1;		
				11p15.5p15.4		
				(180000-7100000) x3;		
				20q13.2		
				(50380000-50640000) x1		
7	23^+1^	Multiple malformations	CNV-seq	69, XXX		
8	21	Multiple malformations	CNV-seq, WES	*ALG1*(NM-019109.4);	AR; Het;	
				c.259G>C (p.G87R)	Paternal;	Novel/VUS(PM2-supporting+BP4)
				c.1327G>A (p.E443K)	Maternal;	Novel/VUS(PM2-supporting)
9	16	Multiple malformations	CNV-seq, WES	No abnormalities		
10	24	Multiple malformations	CNV-seq, WES	No abnormalities		
11	24^+6^	Short limb deformities	CNV-seq, Panel	*FGFR3* (NM-000142); c.742C>T (p.R248C);	AD; Het; De novo;	Reported/P (PMID:7773297)
12	19^+5^	Short limb deformities	CNV-seq, Panel	*FGFR3* (NM-000142); c.742C>T (p.R248C);	AD; Het; De novo;	Reported/P
13	26^+5^	Short limb deformities	CNV-seq, Panel	*FGFR3* (NM-000142); c.835A>T (P.S279C)	AD; Het; De novo;	Reported/P (PMID:17895900)
14	32^+4^	Short limb deformities	CNV-seq, Panel	*FGFR3* (NM-000142); c.1138G>A (p.G380R)	AD; Het; De novo;	Reported/P (PMID:25614871)
15	24	Short limb deformities	CNV-seq, Panel	*FGFR3* (NM-000142); c.1949A>T (p.K650M)	AD; Het; De novo;	Reported/P (PMID:10053006)
16	20	Short limb deformities	CNV-seq, Panel	*COL1A1* (NM-000088); c.1094G>T (p.G365V)	AD; Het; De novo;	Reported/P (PMID:17078022)
17	18^+^	Short limb deformities	CNV-seq, Panel	*COL1A2* (NM-000089); c.3134G>A (p.G1045D)	AD; Het; De novo;	Novel/LP (PS2+PM2-supporting+PP3)
18	22	Short limb deformities	CNV-seq, Panel	*COL1A2* (NM-000089); c.2835+1G>T	AD; Het; Maternal;	Reported/P (PMID:15077201)
19	35	Short limb deformities	CNV-seq, Panel	*COL2A1* (NM-001844); c.3121G>A (p.G1041S)	AD; Het; De novo;	Reported/P (PMID: 17347327)
20	22^+5^	Short limb deformities	CNV-seq, Panel	*COL2A1* (NM-001844); c.2213G>T (p.G738V)	AD; Het; De novo;	Novel/LP (PS2+PM2-supporting+PP3)
21	23	Other skeletal dysplasia	CNV-seq, Panel	*FGFR2* (NM-000141); c.755C>G (p.S252W)	AD; Het; De novo;	Reported/P (PMID:26380986)
22	27	Short limb deformities	CNV-seq, Panel	*RMRP* (NR-003051.3);	AR; Het;	
				n.181G>A	Maternal;	Reported/LP (PMID:27862957)
				n.70G>A	Paternal;	Reported/LP (PMID:12107819)
23	25	Short limb deformities	CNV-seq, Panel	No abnormalities		
24	27^+5^	Short limb deformities	CNV-seq, Panel	No abnormalities		
25	17	Short limb deformities	CNV-seq, Panel	No abnormalities		
26	19^+4^	Short limb deformities	WES	*FGFR3* (NM-000142); c.742C>T (p.R248C);	AD; Het; De novo;	Reported/P
27	24	Short limb deformities	WES	*FGFR3* (NM-000142); c.742C>T (p.R248C);	AD; Het; De novo;	Reported/P
28	19^+5^	Short limb deformities	WES	*FGFR3* (NM-000142); c.742C>T (p.R248C);	AD; Het; De novo;	Reported/P
29	19	Short limb deformities	WES	*FGFR3* (NM-000142); c.742C>T (p.R248C);	AD; Het; De novo;	Reported/P
30	33	Short limb deformities	WES	*FGFR3* (NM-000142); c.1138G>A (p.G380R)	AD; Het; De novo;	Reported/P
31	31^+2^	Short limb deformities	WES	*FGFR3* (NM-000142); c.1138G>A (p.G380R)	AD; Het; De novo;	Reported/P
32	29^+4^	Short limb deformities	WES	*FGFR3* (NM-000142); c.1138G>A (p.G380R)	AD; Het; De novo;	Reported/P
33	28	Short limb deformities	WES	*FGFR3* (NM-000142); c.1138G>A (p.G380R)	AD; Het; De novo;	Reported/P
34	32	Short limb deformities	WES	*FGFR3* (NM-000142); c.1138G>A (p.G380R)	AD; Het; De novo;	Reported/P
35	32	Short limb deformities	WES	*FGFR3* (NM-000142); c.1138G>C (p.G380R)	AD; Het; De novo;	Reported/P (PMID: 7913883)
36	20	Short limb deformities	WES	47, XYY; *COL1A1 *(NM-000088.3); c.2371G>A (p.G791S)	AD; Het; De novo;	Novel/LP (PS2+PM2-supporting+PP3)
37	23^+4^	Short limb deformities	WES	*COL1A1* (NM-000088.3); c.1670G>A (p.G557D)	AD; Het; De novo;	Novel /LP(PS2+PM2-supporting+PP3+PP4)
38	23^+1^	Short limb deformities	WES	*COL1A1* (NM-000088); c.4115A>C (p.N1372T)	AD; Het; De novo;	Novel /LP(PM1+PM2-supporting+PM6+PP3)
39	14^+^	Short limb deformities	WES	*COL1A1* (NM-000088); c.2399G>A (p.G800E)	AD; Het; De novo;	Novel/LP (PM1+PM2-supporting+PM6+PP2+PP3)
40	17^+1^	Short limb deformities	WES	*COL1A2* (NM-000089.3); c.1928G>A (p.G643E)	AD; Het; De novo;	Novel/LP (PM2-supporting+PM6+PP3+PP4)
41	16	Short limb deformities	WES	*COL1A2 *(NM-000089.3); c.2189G>A (p.G730D)	AD; Het; De novo;	Novel/LP (PM2-supporting+PM6+PP3+PP4)
42	13	Short limb deformities	WES	*COL2A1* (NM-001844); c.2303G>T (p.G768V)	AD; Het; Maternal;	Novel/LP (PS2+PM2-supporting-Sup+PP1+PP3)
43	16	Short limb deformities	WES	*DYNC2H1* (NM-001080463);	AR; Het;	
				c.2947-2A>G	Maternal;	Novel/LP (PVS1+PM2-supporting)
				c.7720G>A (p.V2574I)	Paternal;	Novel/LP (PM3+PM2-supporting+PP3)
44	27^+2^	Short limb deformities	WES	*DYNC2H1* (NM-001080463);	AR; Het;	
				c.10606C>T (p.3536*)	Paternal;	Reported/LP (PMID:29068549)
				c.8954T>G (p.V2985G)	Maternal	Novel/LP (PM1+PM2-supporting+PM3+PP3)
45	33	Short limb deformities	WES	*DYNC2H1* (NM-001080463);	AR; Het;	
				c.9182-9185delAGAG (p.E3061fs)	Maternal;	Novel/LP(PVS1+PM2-supporting+PM3)
				c.7495C>G (p.L2499V)	Paternal	Reported/LP (PMID:28492532)
46	16^+6^	Short limb deformities	WES	*DYNC2H1* (NM-001080463);	AR; Het;	
				c.557G>T (p.G186V)	Maternal;	Novel/LP (PM1+PM2-supporting+PM3+PP3)
				c.7643T>C (p.F2548S)	Paternal	Novel /LP (PS1+PM1+PM2-supporting+PP3)
47	14	Short limb deformities	WES	*DYNC2H1* (NM-001080463);	AR; Het;	
				c.8190G>T (p.L2730F)	Maternal;	Reported/LP (ClinVar)
				c.8621delC (p.L2876fs*15)	Paternal	Novel/LP (PVS1+PM2-supporting+PM3)
48	12	Other skeletal dysplasia	WES	*ALPL* (NM-000478.6);	AR; Het;	
				c.1282C>T (p.R428*)	Paternal;	Reported/P (PMID:10636450)
				c.407G>A (p.R136H)	Maternal	Reported/P (PMID:11855933)
49	12	Short limb deformities	WES	*EVC2* (NM-147127.5);	AR; Het;	
				c.871-2A>G	Maternal;	Novel/P (PVS1+PM2-supporting +PM3)
				a deletion of 0.15Mb (chr4:5564145-5710240)	Paternal;	Novel/P (PVS1+PM2-supporting +PM3).
50	13	Other skeletal dysplasia	WES	*GNAI3* (NM-006496); c.119G>T (p.G40V)	AD; Het; De novo;	Novel/LP (PM1+PM2-supporting +PM6+PP3)
51	33^+6^	Short limb deformities	WES	*NPR2* (NM-003995.3); c.1111C>T (p.R371*)	AD; Het; Maternal*;	Reported/LP (PMID:25525159)
52	23	Short limb deformities	WES	No abnormalities		
53	23	Short limb deformities	WES	No abnormalities		
54	25^+4^	Other skeletal dysplasia	WES	No abnormalities		
55	18^+5^	Short limb deformities	WES	No abnormalities		

*AD: Short stature with nonspecific skeletal abnormalities; The phenotype of the mother: short stature (150 cm)

**Fig. 2:**
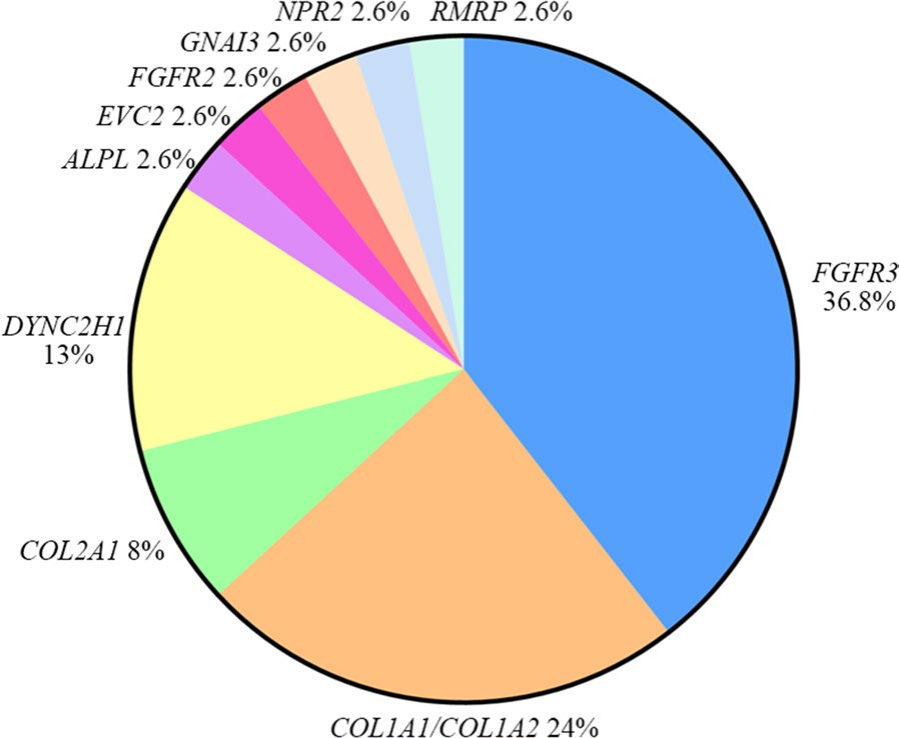
Genes in the present study. Eleven genes were diagnosed in 38 fetuses, including *FGFR3*(15/38), *COL1A1*/*COL1A*2(9/38), *COL2A1*(3/38), *DYNC2H1*(5/38), *ALPL* (1/38), *EVC2*(1/38), *FGFR2*(1/38), *GNAI3*(1/38), *NPR2*(1/38) and *RMRP* (1/38)

To provide a molecular diagnosis, targeted skeletal gene panel sequencing accompanied by CNV-seq was applied to fetuses 11–25, and WES to fetuses 8–10 and 26–55. Thirty-eight cases were diagnosed with hereditary diseases, yielding a diagnostic rate of 79.17% (38/48). Thirty-six causative variants were detected in 11 bone development-related genes, including *FGFR3* (15/38), *COL1A1*/*COL1A2*(9/38), *DYNC2H1*(5/38), *COL2A1*(3/38), *ALPL* (1/38), *EVC2*(1/38), *FGFR2*(1/38), *GNAI3*(1/38), *NPR2*(1/38) and *RMRP* (1/38) (Fig. 2, Table 1). Of these, 18 were novel variants. De novo mutations (DNMs) were identified in 27 (71.05%, 27/38) cases with autosomal dominant (AD) inheritance, compound heterozygotes were identified in eight (21.05%, 8/38) cases with autosomal recessive inheritance, and in the remaining three (7.9%, 3/38) cases with AD inheritance, the variants were inherited from the mother. Detailed information is provided in Table 1.

### Case examples

#### Case 42

**Fig. 3:**
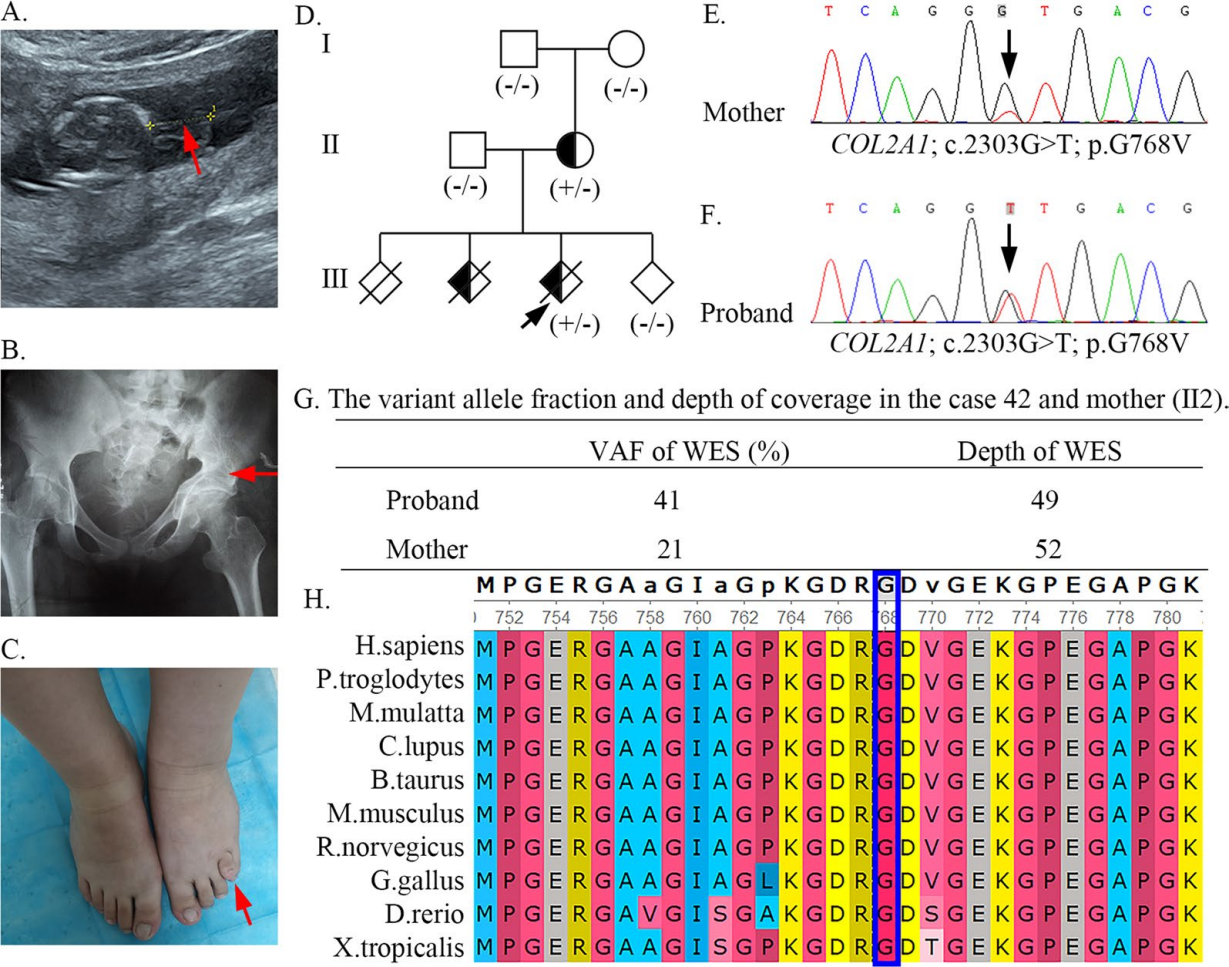
*COL2A1* variant caused severe type 2 collagenopathy in case 42 and mother (II2). **A** The image of US examination of case 42 revealed short limbs. **B**, **C** Clinical photograph of the mother with her pelvis inclined on the left side, shortened left lower limb (before extension osteotomy), and shortened fourth toe in the left foot (arrow). **D** The pedigree map of case 42. **E**, **F** Sanger sequencing of case 42 and the mother (II2). **G** The variant allele fraction and depth of coverage of the *COL2A1* pathogenic variant detected by WES in case 42 and the mother. **H** The variant (*COL2A1* c.2303G > T) was highly conserved across different species

Case 42 was referred to our hospital because the fetus showed short limbs and fetal hydrops evaluated at 14 weeks and 4 days using US examination (Fig. 3A). Pregnancy was terminated at 17 weeks of gestation. The couple’s second pregnancy was evaluated at 22 weeks for suspected abnormal development of fetal limbs (micromelia; US image unavailable) and terminated. WES analysis confirmed that case 42 was heterozygous for the *COL2A1* (NM_001844.4) c.2303G > T variant, resulting in a protein change: p.G768V, consistent with severe type 2 collagenopathy. Sanger sequencing confirmed that the fetus carried the mutation, which was also detected in the mother (the peak of the T allele was much lower than that of the G allele) (Fig. 3E, F). Further detailed clinical assessment of the mother at age 25 revealed mild symptoms compared with those of her fetus, including short stature (height 152 cm, father 175 cm, mother 162 cm), slightly shorter length of the left lower limb than that of the right, and a protruding short fourth toe on her left foot (Fig. 3C). In addition, she showed visual abnormalities, including severe myopia. When she was 1 year old, her left lower limb was shorter than her right lower limb by 2 cm. Her condition seemed to worsen, and she had difficulty walking due to genu varus of the left knee at age 7. Physical examination at age 16 revealed left lower extremity varus deformity, shortening (the length of the right lower limb was 66.8 cm while that of the left lower limb was 58.6 cm), internal rotation deformity of the left leg, and pelvic inclination (Fig. 3B). The patient presented limb claudication and had undergone femoral osteotomy to correct the 8-cm shortening of the left lower extremity. Due to the asymmetrical phenotype and two similar experiences of abortion, we supposed that the mother was affected by mosaicism. For an accurate diagnosis, WES was applied to the mother (II:2), and Sanger sequencing was performed on her parents. The WES results exhibited the mutated allele was present in 11 of 52 (21.2%) reads (Fig. 3G), consistent with the results of Sanger sequencing. *COL2A1* c.2303G > T was not detected in her parents and has not yet been recorded in any database. The novel de novo pathogenic variant (*COL2A1* c.2303G > T) was highly conserved across different species (*Homo sapiens*, *Pan troglodytes*, *Macaca mulatta*, *Canis lupus familiaris*, *Bos taurus*, *Mus musculus*, *Rattus norvegicus*, *Gallus gallus*, *Danio rerio*, and *Xenopus tropicalis*) (Fig. 3H).

#### Case 49

**Fig. 4:**
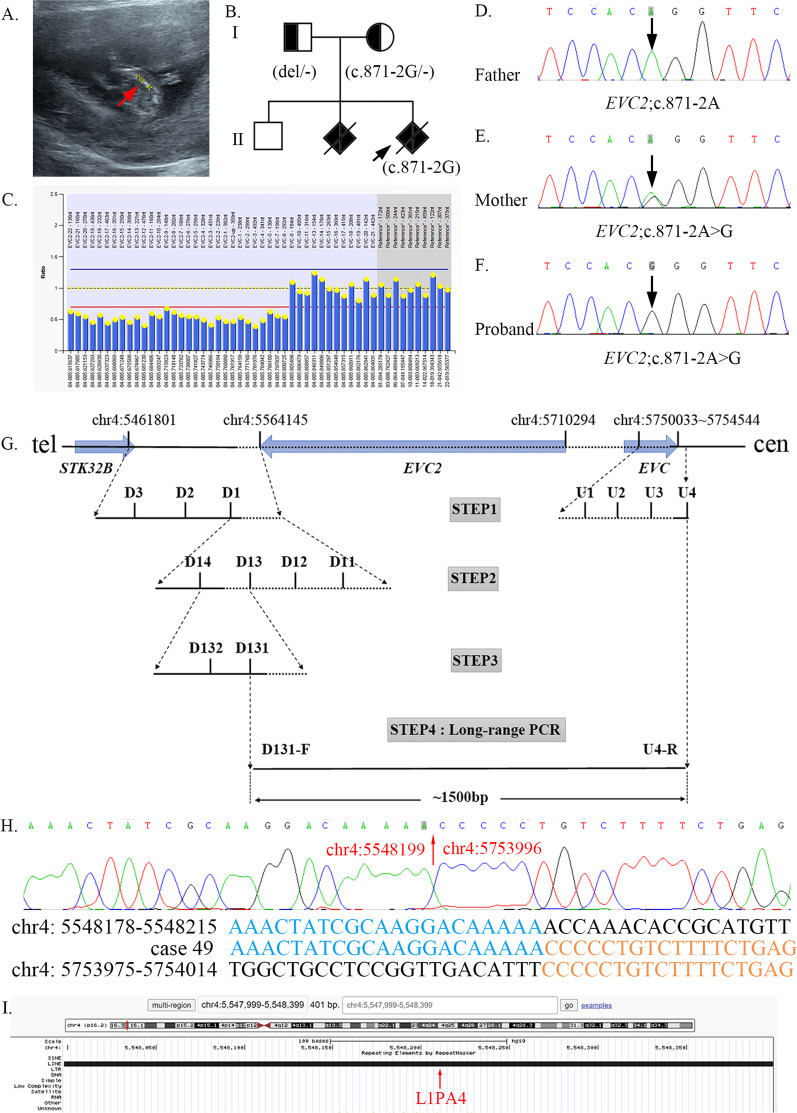
*EVC2* variants caused EVC in case 49. **A** The image of US examination of case 49 revealed short and curved long bones. **B** The pedigree map of case 49. **C** MLPA results of *EVC2* in case 49: a heterozygous deletion of *EVC2*-22 ~ *EVC*-8. **D**–**F** Sanger sequencing showed that the proband had a novel variant inherited from the mother. **G** Schematic presentation of the gross deletion. Arrows indicate genes; Solid horizontal lines indicate retained regions; Broken horizontal lines indicate deleted regions; Multiple primers were designed to be regularly spaced every ~ 25 kb (step 1), ~ 5 kb (step 2), and ~ 1.5 kb (step 3) within breakpoint regions. Long-range PCR with the forward primer of the D131 and the reverse primer of the U4 generates a PCR product of ~ 1.5 kb. **H** The breakpoint sequences of case 49 and Sanger-sequence of the gross deletion. **I** UCSC Genome Browser information around the telomeric breakpoint regions (chr4: 5,547,999–5,548,399)

Due to positive pregnancies of fetal SD, case 49 came to our attention. The pregnancy was terminated because of the abnormal US at 12 weeks of gestation, presenting shortened and curved long bones (Fig. 4A**)**. Two years ago, a scan at 23 weeks of gestation of the first affected fetus exhibited short long bones (< 5standard deviation), polydactylism, and a “common” atrioventricular  valve ring with the absence of the cardiac crux. Compound heterozygous variants (c.871-2A > G and whole *EVC2* gene deletion) in *EVC2* were found in case 49 using WES, inherited from the mother and father, respectively, which were validated using Sanger sequencing and MLPA analysis (Fig. 4C–F). Both variants were PVS1 + PM2-supporting + PM3 and classified as “pathogenic” according to the ACMG guidelines. Collectively, the affected fetuses were diagnosed with Ellis-van Creveld syndrome (EVC, MIM#225500). WES-based CNV analysis revealed heterozygosity at telomeric SNP rs2301856 (chr4:5461801) and centromeric SNP rs899691 (chr4:5754544), suggesting that the deleted region was smaller than 290 kb. To determine the precise size and breakpoints of the gross deletion, we estimated the number of copies (one or two) using qPCR analysis. We performed long-range PCR at 1.5-kb consecutive intervals to amplify the upstream and downstream regions of the *EVC2* gene of the proband. The strategy and results are presented in Fig. 4G, H. Long-range PCR was performed for the affected fetus using the forward primer of D131 and reverse primer of U4, generating a PCR product of 1.5 kb. The aligned sequences revealed a deletion of 205 kb (Chr4:5,548199–5753996) involving the entire *EVC2* gene and a part of the *EVC* gene. To investigate the potential underlying mechanism, the sequences between the two missing boundaries were aligned, and no microhomologous sequence was found. After searching for repetitive elements in the vicinity of the breakpoint, the telomeric breakpoint was found to be in a long interspersed nuclear element (LINE, L1PA4) belonging to the L1 family (Fig. 4I**)**.

#### Case 50

**Fig. 5:**
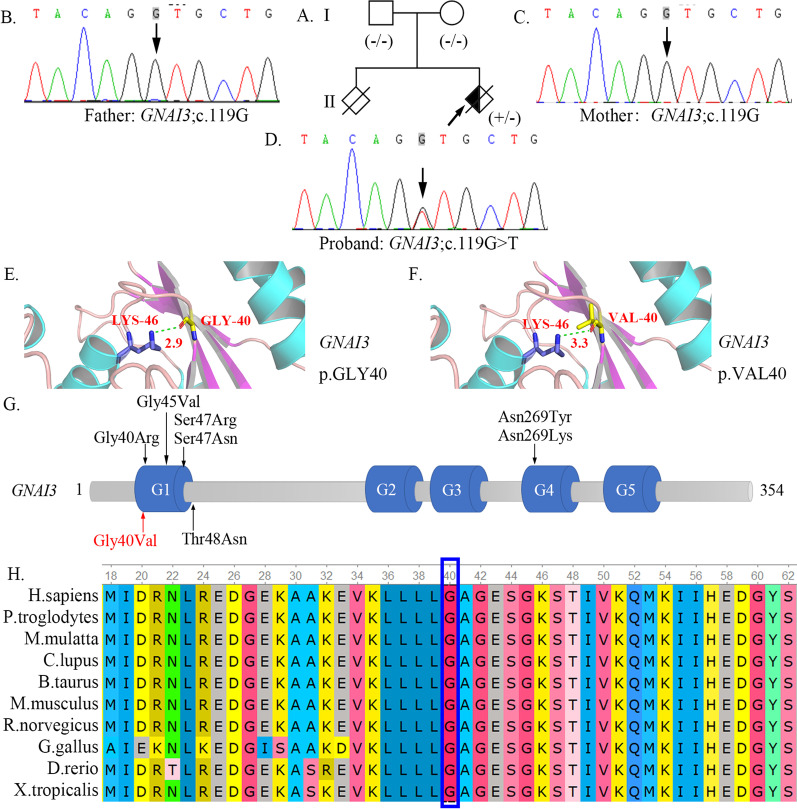
*GNAI3* variant caused auriculocondylar syndrome (ACS) in case 50. **A** The pedigree map of case 50. **B**–**D** Sanger sequencing showed that the proband had a de novo variant. **E**, **F** The 3D molecular structure of Gnαi3. The magnified views of the wild-type Gly40 (**E**) and mutant Val40 (**F**) are shown respectively. The H-bonds are shown as green dashed lines, and H-bond distances (Å) are shown in red numbers **G** Gnαi3 domains are depicted in blue: boxes G1–G5. The variant reported here is in red, and previously described variants are in black. **H** Protein alignment showing conservation of residues *GNAI3* p.Gly40Val across multiple species. This mutation occurred at evolutionarily conserved amino acid positions

Case 50 was admitted to our hospital at 13 weeks of gestation and was diagnosed as micrognathia by US (mandibular length: 8 mm, biparietal diameter: 27 mm). Besides, oligohydramnios was found in case 50 (the single deepest pocket of amniotic fluid was 40 mm). WES demonstrated a heterozygous variant, c.119G > T (p.G40V), in the *GNAI3* gene. The verification results of the parents suggested that the variant was a de novo mutation (Fig. 5B–D**)**. The novel variant was classified as “likely pathogenic” based on the interpretation of PM1 + PM2-supporting + PM5 + PM6 + PP3 according to the ACMG guidelines. Structural modeling showed that glycine 40 was located in the random coil structure of the protein secondary structure and formed a hydrogen bond with lysine 46, which possessed a bond length of 2.9 Å (Fig. 5E). After changing to valine, the hydrogen bond was slightly lengthened to 3.3 Å (Fig. 5F). Glycine 40 of Gnai3 is highly conserved among species (*Homo sapiens*, *Pan troglodytes*, *Macaca mulatta*, *Canis lupus familiaris*, *Bos taurus*, *Mus musculus*, *Rattus norvegicus*, *Gallus gallus*, *Danio rerio*, and *Xenopus tropicalis*) (Fig. 5H). In line with the aforementioned results, the etiology of case 50 was attributed to the mutation in *GNAI3*.

## Discussion

In this study, molecular testing of 55 cases yielded a total detection rate of 81.82% (45/55), which was slightly higher than 76.67% (23/30), 64% (35/55), 24% (8/34), and 15% (10/65) reported in previous studies on fetal bone abnormalities in recent years [3, 8–10]. The detection rate is affected by many factors, such as the cohort size, study inclusion criteria, and methods. The discrepancy in the detection rates may be explained by sample differences. In our study, most of the samples were fetal abortion tissue samples, which reflected the severity of the phenotypes. The diagnosis involved seven cases with chromosomal abnormalities and 38 with gene variants. Notably, the diagnostic rate of chromosomal abnormalities in fetuses with multiple malformations in our study was as high as 70% (7/10), comparable to 50% (1/2) and 62.5% (5/8) reported by Jin Han et al. [11] and Liu et al. [8] respectively. Nonetheless this observation must to be validated in a larger cohort. In addition, compared with fetuses with only skeletal malformation, cases with multiple malformations had the highest karyotype and chromosomal microarray analysis (CMA) detection rates according to Fu et al. [12] (karyotype 4.9% vs. 26.9%, and CMA 5.9% vs. 12.6%). These findings suggest that CNV analysis is an accurate and cost-effective method for identifying the etiology of SD, especially in affected fetuses with multiple malformations. Variants, of which 18 were novel, were detected in 38 cases in this study, at a rate of 79.17% (38/48). These results enrich the spectrum of genetic variants associated with fetal SD. The rate of DNMs was 71.05% (27/38), while a rate of 60% (21/35) was reported in a related study [9], which re-emphasized the importance of prenatal diagnosis and gene analysis for SD-afflicted fetuses.

The most common nosology groups in our study were the FGFR3 chondrodysplasia group (15/38, 39%), osteogenesis imperfecta and decreased bone density group (9/38, 24%), and ciliopathies with major skeletal involvement (5/38, 13%), consistent with the results of other studies [13, 14]. Furthermore, *FGFR3* variants are the most common cause of fetal SD. In our cohort, cases with the *FGFR3* c.1138G > A (6/38, 15.79%) and c.742C > T (6/38, 15.79%) variants accounted for one-third of the total cases.

Mosaicism may have been more common than previously estimated. In a 2019 study by Cao et al. [15], disease-related mosaic variants were present in 1.5% of probands, and 0.3% were parental mosaic variants in cases with a definite molecular diagnosis based on 12,000 whole-exome data. In our study, the mother of case 42 had two fetuses affected by severe SD. She carried a novel somatogonadal mosaic variant, *COL2A1* c.2303G > T, which was detected using WES (21.2% mosaicism in her peripheral blood DNA) and confirmed using Sanger sequencing. It is worth noting that the abnormal skeletal symptoms in the mother were asymmetrical. Her left lower limb was shorter than her right, and a protruding short fourth toe was also present on her left foot, while her right lower limb and foot were normal. A father with somatogonadal mosaicism of *COL2A1* c.1403G > A (22% mosaicism in blood DNA) exhibited average stature (height 173 cm) and had two affected fetuses with severe type 2 collagenopathy carrying the heterogynous mutation identified by Morrison et al. [16]. Yamamoto et al. described a similar phenomenon in mild type 2 collagenopathy with paternal somatogonadal mosaicism [17]. Based on these findings, we suspected that there were many complex mechanisms between the phenotype and mosaicism levels, not just the threshold effect [16]. Moreover, it has been suggested that because of the differences in the time and region of somatic mosaicism, the clinical phenotype presented individuation [18]. The intrafamilial recurrence rate in gonosomal mosaicism was high [19], so it is of great significance to detect gonosomal mosaicism and provide recommendations for performing prenatal diagnosis in subsequent pregnancies.

Based on positive family history and the US findings, the clinical diagnosis of EVC syndrome can also be made for the first affected fetus of family 49 [20]. EVC syndrome is a chondral and ectodermal dysplasia characterized by short stature, thoracic hypoplasia, dysplastic fingers, teeth, nails, and congenital heart disease (occurring in approximately 60% of cases), especially atrioventricular septal defect and single atrium [21]. In addition, patent ductus arteriosus in fetuses with EVC syndrome has previously been reported [20]. A common atrioventricular valve with the absence of the cardiac crux was found at 22 weeks of gestation in the first affected fetus of family 49 using the three-dimensional US. However, the second affected fetal intracardiac structures could not be revealed by the US at 12 weeks of gestation. Our findings on the affected fetuses were consistent with the existing literature. A novel compound heterozygous variant (c.871-2A > G) and a gross 205 kb deletion in *EVC2* were identified in case 49. Finally, we sought to define the boundaries of the deletion and decipher the underlying mechanism. Using a qPCR walking strategy to sequence the breakpoint junction, the telomeric breakpoint was considered to be derived from repeating elements that overlapped with LINE (L1PA4). Rearrangement of breakpoints may result from distinct mutational mechanisms: (1) homologous recombination, (2) non-homologous end joining, (3) microhomology-mediated replication-dependent recombination, (4) LINE-1-mediated retrotransposition, and (5) telomere healing [22]. LINE-1 sequences constitute a significant factor in genome arrangements [23]. These regions may be fragile and prone to chromosomal abnormalities.

Previous studies have implicated variants of the *GNAI3* gene in auriculocondylar syndrome (ACS, MIM#602483), a rare AD disorder characterized by micrognathia, mandibular condyle hypoplasia, and question mark auricular malformation [24]. A novel de novo variant, c.119G > T (p.G40V) in *GNAI3,* was identified in case 50 using WES analysis because of “micrognathia” at 13 weeks of gestation detected using US examination. To the best of our knowledge, this is the fourth fetus afflicted with ACS with a *GNAI3* mutation. Liu et al. summarized the clinical features of the other three fetuses, suggesting that severe micrognathia and mandibular hypoplasia accompanied by polyhydramnios were prenatal indicators of ACS [24]. Amniotic fluid was normal in case 50 (amniotic fluid volume was 40 mm) in the present study. A possible explanation for this is the incomplete expression of the phenotype in the fetus in the first trimester. In the future, prenatal characteristics must be identified in more affected fetuses to promote ACS awareness. To date, only seven variants of *GNAI3* have been reported [24], and all eight variants were missense, including p.Gly40Val reported in the present study (Fig. 5G). Gnai3, encoded by *GNAI3,* consists of five highly conserved motifs (G1–G5) that are involved in the rat sarcoma superfamily and G α protein binding to guanosine diphosphate (GDP)/guanosine triphosphate (GTP) [25]. Most variants (7/8, 87.5%) were located in the G1 or G4 box, and the p.Gly40Val variant was located in the G1 box (Fig. 5G). Although the p.Gly40Val variant did not disrupt the overall structure of the protein as demonstrated using structural modeling, it may indirectly disrupt GDP/GTP binding [26].

Our study has some limitations. First, our sample size was relatively small, and most tissue samples were derived from fetal abortion. Thus, the results of a larger sample size would be more objective and accurate regarding the diagnosis rate of fetal SDs. Second, different tissues of the mother of case 42 were not collected to explore the relationship between the mosaicism level and asymmetrical phenotype. Third, because case 50 was detected in the first trimester, we could not obtain more useful clinical information, except for micrognathia. In addition, further functional studies should be performed to analyze the effects of these variants, especially those of the novel and VUS variants.

## Conclusions

In summary, the detection rate in our study was 81.82% (45/55), and the detection rate of CNV analysis in multiple malformations was 70% (7/10), indicating that CNV analysis is essential in SD fetuses with multiple malformations. In addition, 38 different variants were identified, of which 18 were novel. Moreover, we demonstrated gonosomal mosaicism in a patient with asymmetrical symptoms and confirmed the breakpoints of a novel gross deletion. Our study is informative for genetic counselling and future pregnancies of affected families and extends the clinical features and genetic spectrum of fetal SD.

## Supplementary Information

**Additional file 1. Table S1:** 811 genes of a genetic skeletal disease panel; **Table S2:** Primer sequence in this study; **Table S3:** Summary of clinical information and molecular results for 55 cases with skeletal abnormalities.

**Additional file 2.** Images of US examination of some fetuses in this study.

## Data Availability

The original data belonging to this study are available from the corresponding author upon request.

## Abbreviations

SD: Skeletal dysplasia
US: Ultrasound
CNV-seq: Copy number variation sequencing
WES: Whole exome sequencing
QF-PCR: Quantitative fluorescent-PCR
OMIM: Online Mendelian inheritance in man
NGS: Next generation sequencing
1000G: 1000 genomes project
G nomAD: Genome aggregation database
HGMD: Human genomic mutation database
ACMG: American College of Medical Genetics and Genomics
MLPA: Multiplex ligation-dependent probe amplification
DNMs: De novo mutations
AD: Autosomal dominant
EVC: Ellis-van creveld syndrome
LINE: Long interspersed nuclear element
CMA: Chromosomal microarray analysis
ACS: Auriculocondylar syndrome
GDP: Guanosine diphosphate
GTP: Guanosine triphosphate
