# Supplementary material for: Genetic analysis of 55 cases with fetal skeletal dysplasia
Source: Orphanet J Rare Dis. 2022 Nov 9;17:410. doi: 10.1186/s13023-022-02559-4 (PMC9648031; doi:10.1186/s13023-022-02559-4)
Supplement: Supplementary file 2 — Additional file 2. Images of US examination of some fetuses in this study. [file 13023_2022_2559_MOESM2_ESM.pdf]

Images of US examination of some fetuses in this study.

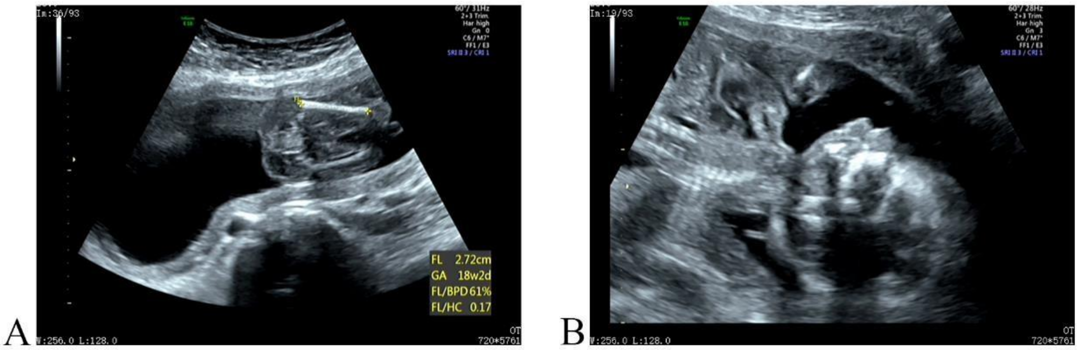

Additional file 2-Fig. S1: Ultrasound figures show shortened femur (2.72cm, -3.03SD) (A) and slightly shortened nose bone and receding jaw (B) in case 1 with Trisomy-18.

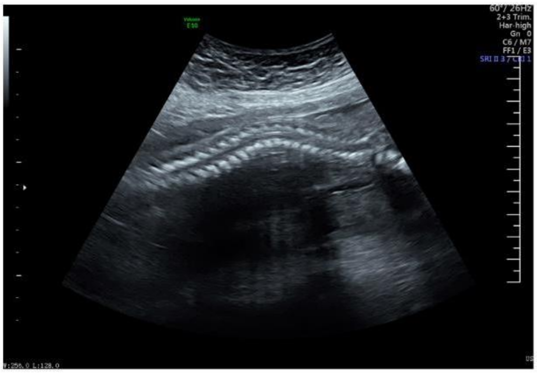

Additional file 2-Fig. S2: An ultrasound figure shows spinal fissure in case 6 (46, XX; Xp22.33p22.12 (2700000-19680000) x1;11p15.5p15.4 (180000-7100000) x3;20q13.2 (50380000-50640000) x1).

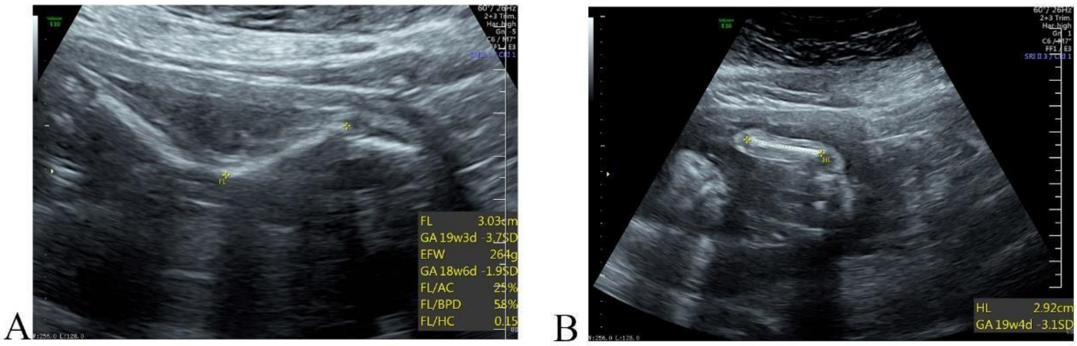

Additional file 2-Fig. S3: Ultrasound figures show shortened femur (3.03cm, -3.7SD) (A) and shortened humerus (2.92cm, -3.1SD) (B) in case 7 (69, XXX).

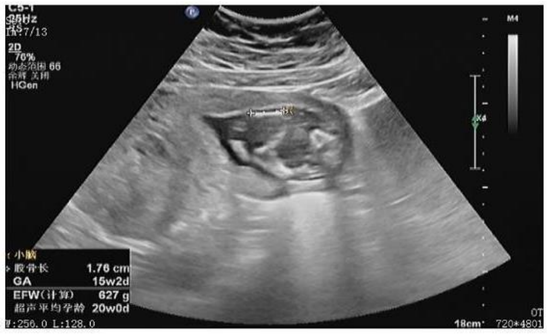

Additional file 2-Fig. S4: An ultrasound figure shows shortened femur (1.76cm, -7.56SD) in case 8 (*ALG1* c.259G>C and c.1327G>A).

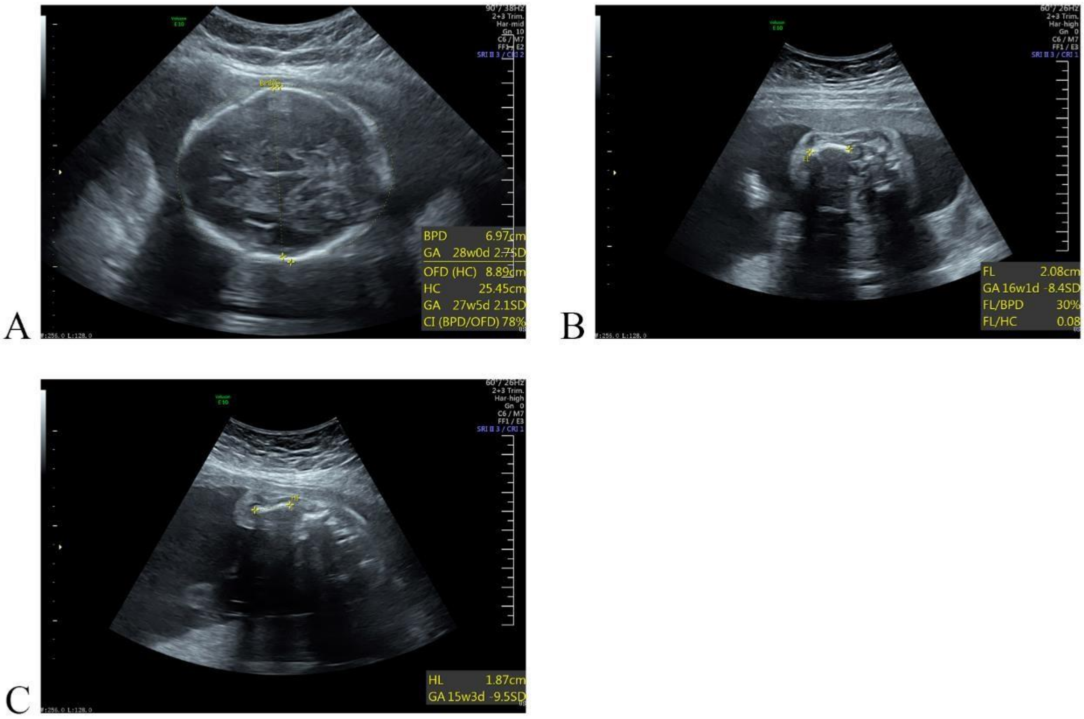

Additional file 2-Fig. S5: Ultrasound figures show a large biparietal diameter (6.97cm, 2.7SD) (A), shortened and curved femur (2.08cm, -8.4SD) (B), and shortened humerus (1.87cm, -9.55SD) (C) in case 11 (*FGFR3* c.742C>T).

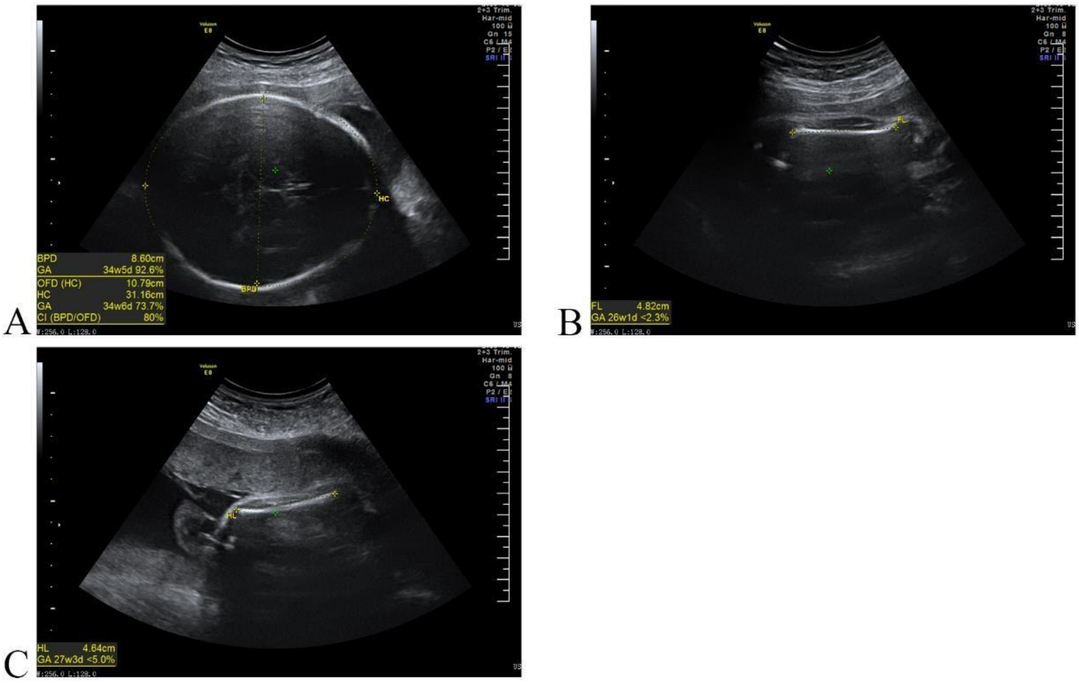

Additional file 2-Fig. S6: Ultrasound figures show a normal biparietal diameter (8.60cm, 1.03SD) (A), shortened and curved femur (4.82cm, -4.76SD) (B), and shortened humerus (4.64cm, -3.54SD) (C) in case 14 (*FGFR3* c.1138G>A).

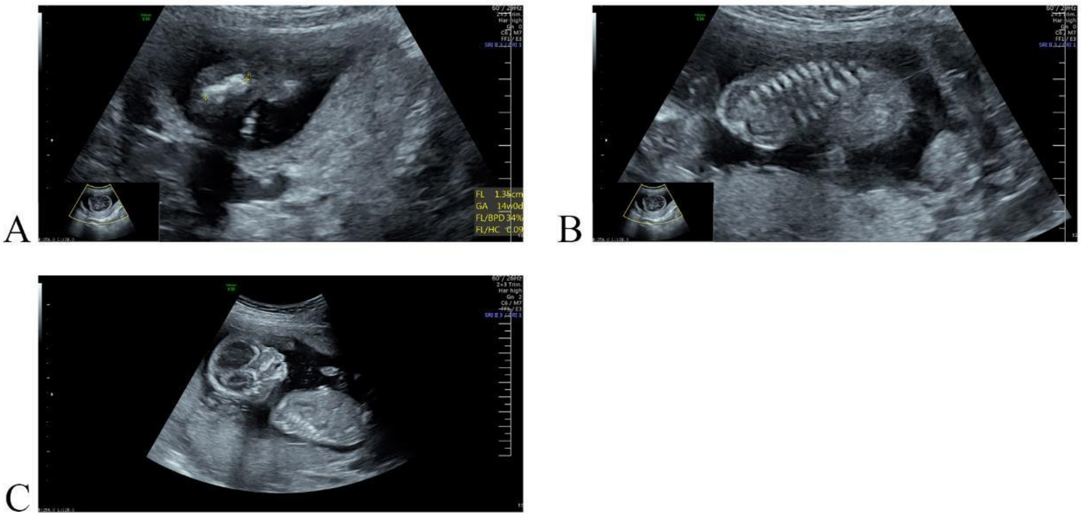

Additional file 2-Fig. S7: Ultrasound figures show shortened femur with angular deformity (1.35cm, -6.05SD) (A), wavy-shaped ribs (B), and low calcification of the skull (C) in case 17 (*COL1A2* c.3134G>A).

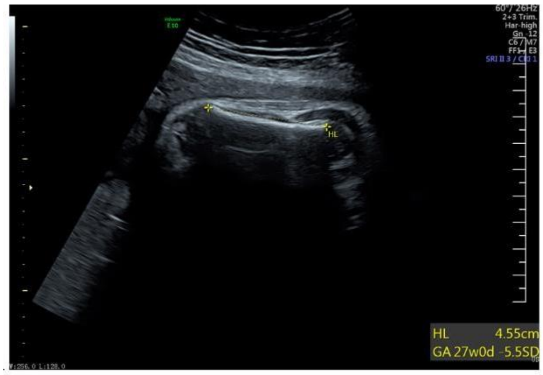

Additional file 2-Fig. S8: An ultrasound figure shows shortened femur (4.55cm, -5.5SD) in case 22 (*RMRP* n.181G>A and n.70G>A).

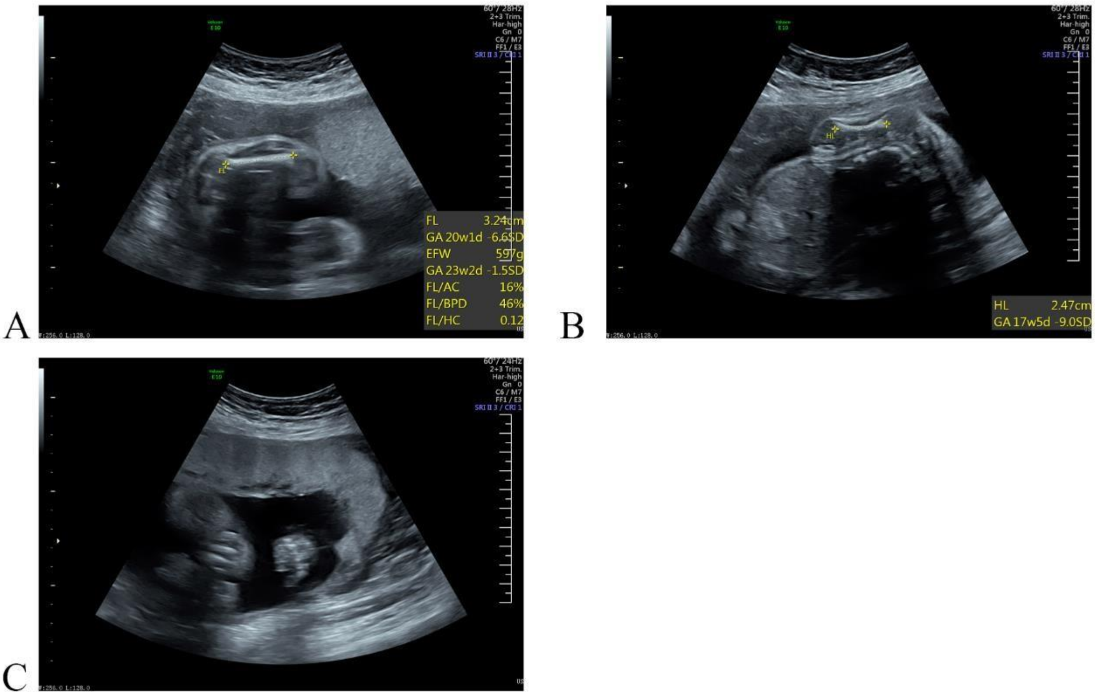

Additional file 2-Fig. S9: Ultrasound figures show shortened femur with angular deformity (3.24cm,

-6.6SD) (A), shortened and curved humerus (2.47cm, -9.0SD) (B), and polydactyly (C) in case 44 (*DYNC2H1* c.10606C>T and c.8954T>G).

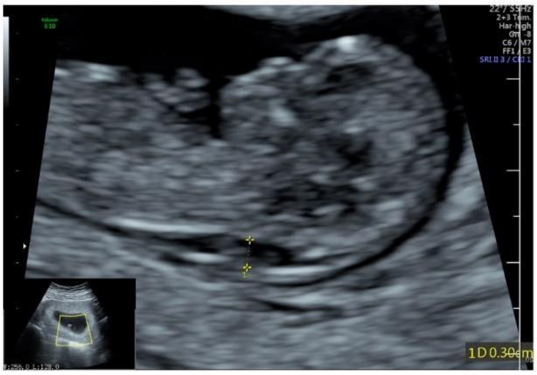

Additional file 2-Fig. S10: An ultrasound figure shows poor ossification of the whole skeletal system in case 48 (*ALPL* c.1282C>T and c.407G>A).

A

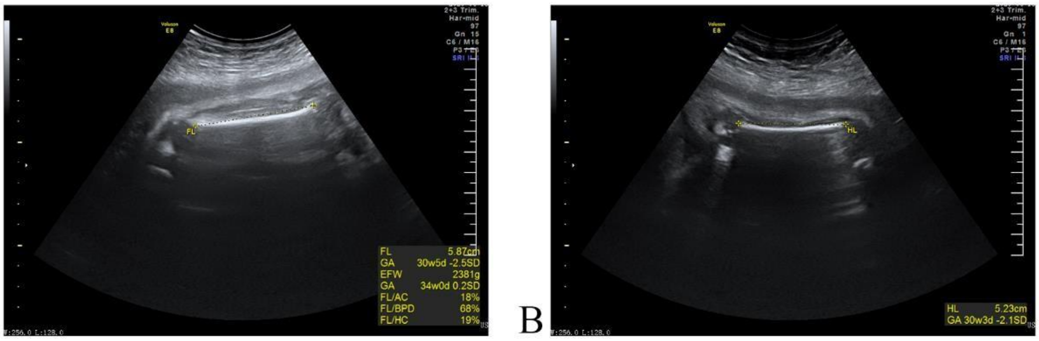

Additional file 2-Fig. S11: Ultrasound figures show shortened femur (5.87cm, -2.5SD) (A) and shortened humerus (5.23cm, -2.1SD) (B) in case 51 (*NPR2* c.1111C>T).

A

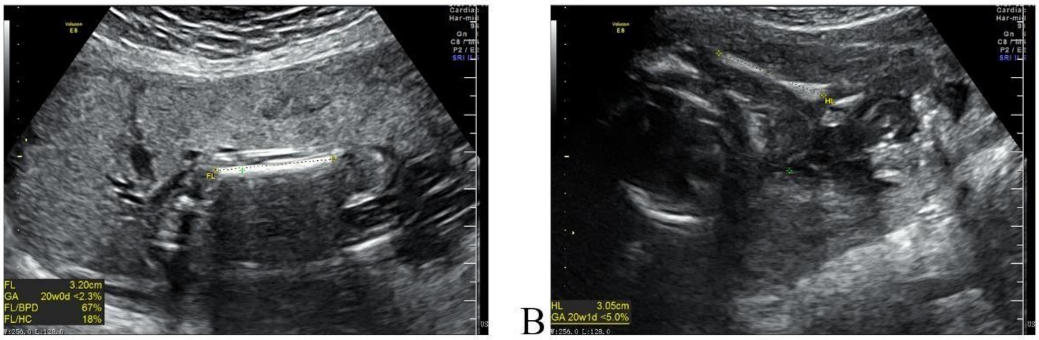

Additional file 2-Fig. S12: Ultrasound figures show shortened femur (3.20cm, -3.46SD) (A) and shortened humerus (3.05cm, -3.56SD) (B) in case 53.

*SD: standard deviation
